# The Responses of the Quantitative Characteristics of a Ramet Population of the Ephemeroid Rhizomatous Sedge *Carex physodes* to the Moisture Content of the Soil in Various Locations on Sand Dunes

**DOI:** 10.1155/2014/120186

**Published:** 2014-06-19

**Authors:** Buhailiqiemu Abudureheman, Huiliang Liu, Daoyuan Zhang, Kaiyun Guan, Yongkuan Zhang

**Affiliations:** ^1^Key Laboratory of Biogeography and Bioresource in Arid Land, Xinjiang Institute of Ecology and Geography, Chinese Academy of Sciences, Urumqi 830011, China; ^2^University of the Chinese Academy of Sciences, Beijing 100049, China; ^3^Turpan Eremophytes Botanical Garden, Chinese Academy of Sciences, Turpan 838008, China; ^4^Wuhan Botanical Garden, Chinese Academy of Sciences, Wuhan 430074, China

## Abstract

In this study, the soil moisture content was measured, and the quantitative characteristics of this sedge species were compared. The phenotypic plasticity of each parameter and the linear regression relationships were analyzed. The results showed that the soil moisture content was significantly affected by location, soil depth, and sampling date. The aboveground biomass, underground biomass, biomass density, and population density at the peak were significantly higher than elsewhere on the dune. However, the morphological plasticity index of the quantitative characteristics was higher at the base and middle of the dune. When the soil moisture content decreased, the underground biomass and ramet biomass density increased. The aboveground and underground biomasses were strongly negatively correlated, but the ramet height and aboveground biomass were strongly positively correlated. These results indicated that the soil water content significantly affected the clonal growth of* C. physodes*. The responsiveness of* C. physodes* may be adaptive when the soil resource supply is low. The strong morphological plasticity of the species appears to be ecologically important for the maintenance and dominance of this species in the dune habitat.

## 1. Introduction

Ephemeral plants are an important component of the vegetation in desert ecosystems, and their germination and growth are especially sensitive to changes in water and temperature [1]. The amount and distribution of rainfall regulates the emergence of ephemeral plant species, thereby influencing species richness, composition, and plant density [2]. Recently, however, increasing temperatures have continued to cause an alarming evaporation of the available soil water [3]. Plants are plastic and can adjust to changes in the climate by altering their morphology in response to the environment, with potential effects on resource acquisition [4].

Desert vegetation includes a number of clonal plants. The desert environment is characterized by water shortages, intense evaporation, and nutrient-deficient soil [5]. The biomass allocation patterns and morphological plasticity of clonal plants are crucial adaptations to a heterogeneous environment [6]. Morphological plasticity allows the plastic placement of ramets through clonal integration to enhance the exploitation of heterogeneously distributed resources [7, 8]. Clonal integration can modify biomass allocation to ensure that relatively more biomass is allocated to the organs (roots or leaves), which play the most valuable roles in the acquisition of more abundant resources [9]. Extensive studies have focused on the relationship between plants and soil; however, few reports were performed to investigate the morphological plasticity of ephemeroid plants in response to water deficiency in desert environment. Ephemeroids are perennial herbaceous plants and appear immediately following snow melt. Their fruits ripen rapidly in 40 to 60 d. The aboveground parts perish, but the underground organs remain alive and new individual can be produced on the next spring from underground buds or from seeds.* Carex physodes* is an ephemeroid rhizomatous sedge species. In the Gurbantunggut Desert, rainfall is frequent but limited (89.8% of rainfall occurrences measure less than 5 mm), creating a patchy distribution of soil moisture at various positions on sloping terrain [10]. This species only occurs on mobile semifixed or fixed sand dunes facing windward [11] and often forms a single dominant species community [12]. During spring, the season of high winds and sandstorms,* C. physodes* occurs in dense stands that are distributed in various geomorphic positions and shows a preference for the top of the dune. Accordingly,* C. physodes* can be used to fix and stabilize sand [12, 13]. At present, information about this species is limited to its distribution and growth characteristics in its natural habitat [12–14]. In view of the widespread abundance of this species throughout the desert, the following questions were evaluated: (1) does a heterogeneously distributed and limited soil water supply cause plastic responses in this species due to its effect on the growth of the ramets? (2) Does clonal integration enhance compensatory growth and biomass allocation to the underground and aboveground parts of the plant?

In this study, ramet height, aboveground and underground biomass, ramet biomass density, and ramet population density of* C. physodes* at the base, middle, and peak of sand dunes were measured. The linear regression relationships between various quantitative characteristics and phenotypic plasticity of each parameter on each sampling date were analyzed. This research will shed light on the ecological adaptive strategies of the clonal integration of the ephemeroid rhizomatous species* C. physodes* to the sparse precipitation in the desert. Such information could be useful for desert restoration and rational utilization of resources.

## 2. Materials and Methods

### 2.1. Study Area and Species

The Gurbantunggut Desert is located in the center of the Junggar Basin in Xinjiang. It is the second largest desert in China, with an area of 48,800 km^2^ [15]. The mean annual temperature is 6–10°C, with the maximum temperature exceeding 40°C in July. The mean humidity is approximately 50–60%. The mean annual precipitation is approximately 79.5 mm, with 29% of the total precipitation occurring in April and May [16]. The mean potential annual evaporation is 2607 mm, 20–30 times greater than the precipitation. The climate of the area is typical of inland arid zones. The minimum temperature is −40°C, and snow accumulates to a depth of 20 cm in winter. The mean temperature and precipitation are 13.6°C and 24.7 mm, respectively, in April and 19.4°C and 19.1 mm, respectively, in May (climatological data from the Cainan Oil Station of the Gurbantunggut Desert for 2006–2012). The rainfall in winter and spring creates favorable conditions for the growth of ephemeral plants [12].

**Figure 1:**
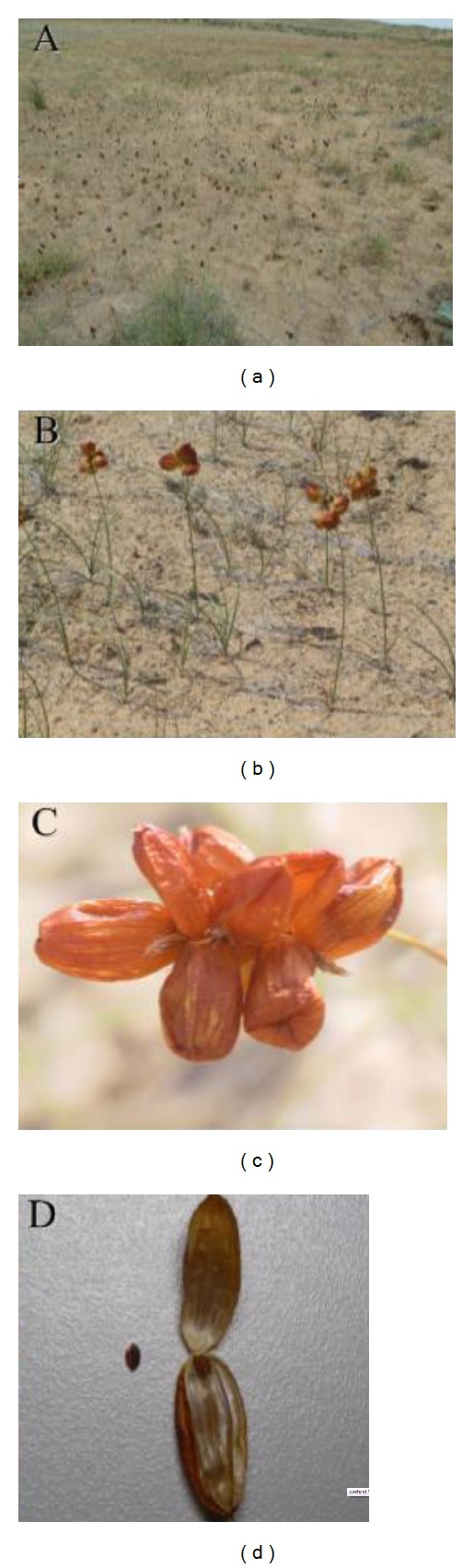
The habitat and morphology of* Carex physodes* in Gurbantunggut Desert. Habitat (a); ramet (b); infructescence (c); and fruit and seed (d).

*Carex physodes* is an ephemeroid sedge, 15–35 cm tall (Figures 1(a) and 1(b)). It reproduces both asexually by underground rhizomes and sexually by seeds. The aboveground parts of this species die in summer, and the underground parts produce new individuals from underground buds when the snow melts during the next spring. The plant has compact inflorescences with androgynous flowers (Figures 1(b) and 1(c)). Flowering and fruiting occur from April to May. The utricles are globose or elongate, conical or cylindrical, and brown or scarious. The seeds are spherical, biconvex, and light brown (Figures 1(c) and 1(d)). However, the germination rate is less than 5%, and seedlings are rarely observed in the wild (unpublished data). Accordingly, the regeneration of the population depends primarily on the clonal growth of underground rhizomes.

### 2.2. Methods

Three sites in the Gurbantunggut Desert were selected (site 1, 44°59′82′′N, 88°23′20′′E; site 2, 45°00′78′′N, 88°22′94′′E; site 3, 44°57′02′′N, 88°22′85′′E). At each site, three sand dunes oriented in a NW-SE direction were selected. On each sand dune, three locations were established at a scale of 5 m × 20 m at the base, middle, and peak of the dune. Five 0.5 m × 0.5 m quadrats were placed at each location. Random samples were taken on five sampling dates (April 19th, May 1st, May 15th, June 2nd, and June 23rd) separated by 10–15 d intervals. Soil samples from each of the three locations on each sand dune were taken at depths of 0–5 cm, 5–10 cm, 10–15 cm, and 15–20 cm (with four replicate samples at each depth) on each sampling date and were dried in an oven at 85°C for 24 h. The height, number of ramets, the aboveground and underground biomasses, and the whole biomass of each ramet were measured in each quadrat on each sampling date. The biomass was determined after drying in an oven at 85°C for 24 h and was measured with a precision balance (to the nearest 0.01 g) in the laboratory.

### 2.3. Statistical Analysis

All statistical analyses used SPSS version 16.0. A one-way ANOVA was used to determine the effects of location (i.e., base, middle, and peak), sampling date, and soil depth on the soil moisture content and to determine the effects of location and sampling date on the quantitative characteristics (ramet height, aboveground and underground biomasses, ramet biomass density, and ramet population density) of* Carex physodes*. A two-way ANOVA was used to examine the effects of location, sampling date, and their interactions on the different quantitative characteristics. A three-way ANOVA was used to evaluate the effects of location,   sampling date, soil layer, and their interactions on the soil moisture content. Multiple comparisons with Bonferroni corrections were performed to determine the differences in different quantitative characteristics within sampling dates and locations (*P* < 0.05). Plasticity indices (PI) were calculated to evaluate the effects of sampling date and location on the various quantitative characteristics. An index of phenotypic plasticity (PI) ranging from 0 to 1 was calculated for each variable. This index was equal to the difference between the minimum and the maximum values of the various quantitative characteristics, at the three locations on the sampling dates, divided by the maximum value [17]. The PI was defined to indicate the variability of the different quantitative characteristics on the three sand dunes on different sampling dates. A linear regression was used to analyze the relationship between the various quantitative characteristics. A Pearson correlation coefficient (*r*) was calculated to evaluate the significance of the relationship between the various quantitative characteristics. The figures were drawn with SigmaPlot version 12.0.

## 3. Results

### 3.1. Comparisons of Soil Moisture Content

The three-way ANOVA showed that the soil moisture content was significantly affected by the location, the sampling date, the soil depth, and the interactions between and among them. However, the interactions involving the three factors did not significantly affect the soil moisture content (Table 1).

**Figure 2:**
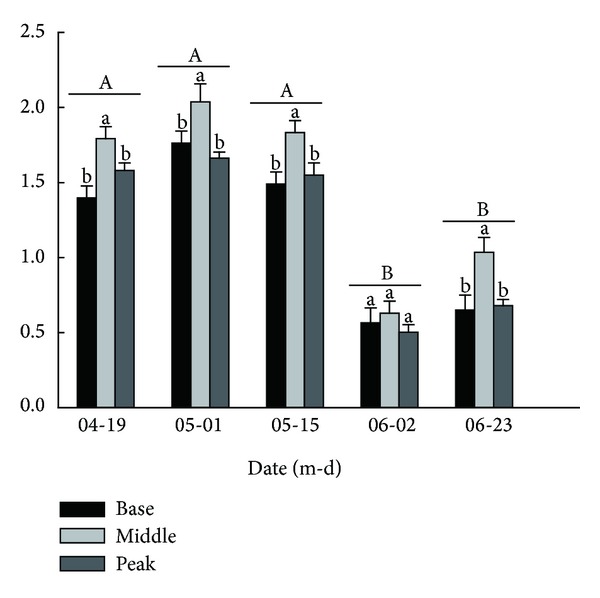
The comparison of the soil moisture content of three locations at different sampling dates (% ±S.E). Different lowercase letters indicate a significant difference among locations at the same sampling date and at the same soil depth (*P* < 0.05). Different uppercase letters indicate a significant difference between different sampling dates at the same soil depth and at the same location.

The comparisons among locations showed that the soil moisture content at the middle of the dune was significantly higher than that at the base or peak of the dune on all the sampling dates except June 2nd (*P* < 0.05), but there were no significant differences between the peak and base of the dune (*P* > 0.05). There were no significant differences among the soil moisture values for April 19th, May 1st, and May 15th (*P* > 0.05) (Figure 2). On June 2nd, a date chosen to represent early summer, the temperature increased. As a result, the soil moisture content reached its lowest value after the melting of the snow. The soil moisture content was significantly lower at a depth of 0–5 cm than at any other depth (data not shown). After the snow has melted, this layer becomes dehydrated as a result of heating and evaporation, and a dry soil layer forms.

### 3.2. Dynamic Curves of Ramet Height of* C. physodes* at Different Locations

**Figure 3:**
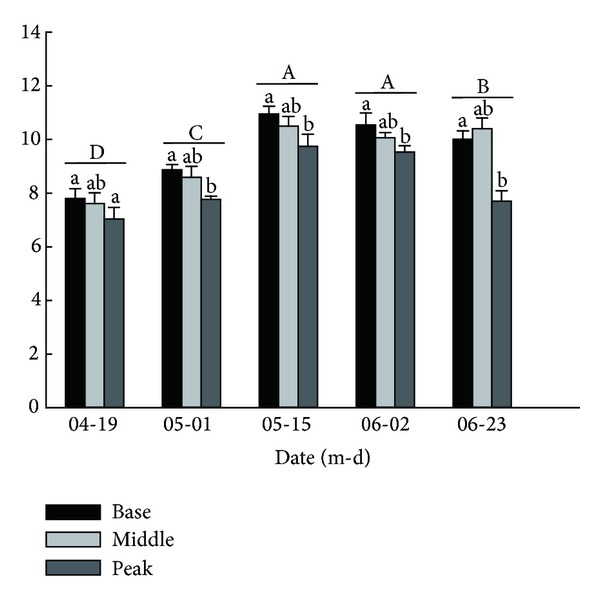
Dynamic curves of ramet height of* Carex physodes* at different locations on different sampling dates. Different lowercase letters indicate a significant difference of ramet height among three locations at the same sampling date (*P* < 0.05). Different uppercase letters indicate a significant difference among sampling dates at the three locations (*P* < 0.05).

The two-way ANOVA showed that the ramet height was significantly affected by the location and sampling date but not by their interaction (Table 2). From mid-April to mid-May, the ramet height increased in each location, with the maximum height achieved in mid-May. The ramet height then decreased slightly. Significant differences were found among all sampling dates except May 15th and June 2nd (*P* < 0.001). The ramet height at the base location was significantly higher than that at the middle and peak locations (*P* < 0.05) except on June 23rd (Figure 3).

### 3.3. Biomass Differences and Population Density between Ramets Growing at Different Locations on Dunes

The two-way ANOVA showed that the aboveground biomass, the underground biomass, and the ramet biomass density were significantly affected by location (*P* < 0.05), sampling date (*P* < 0.001), and their interaction (*P* < 0.001) (Table 2). However, the ramet population density was only significantly affected by the location (*P* < 0.001), not by the sampling date or their interaction (*P* > 0.05) (Table 2).

**Figure 4:**
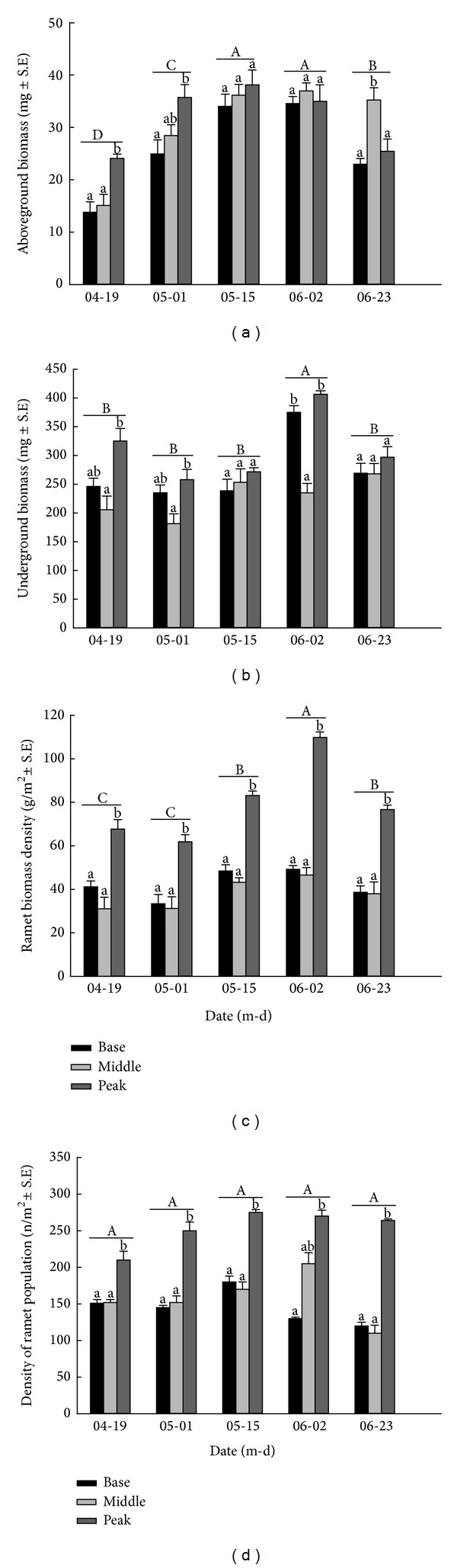
Dynamic curves of aboveground biomass (a), underground biomass (b), ramet biomass density (c), and ramet population density (d) of* Carex physodes* at different locations on different sampling dates. Different lowercase letters indicate a significant difference of ramet height among three locations at the same sampling date (*P* < 0.05). Different uppercase letters indicate a significant difference among sampling dates at the three locations (*P* < 0.05).

The aboveground biomass was higher at the peak than at the base and middle prior to May 15th. Thereafter, the aboveground biomass persisted at the middle location, whereas the peak and base aboveground biomass decreased. The aboveground biomass differed significantly among locations on April 19th, May 1st, and June 23rd (*P* < 0.05) (Figure 4(a)). The underground biomass (except on June 2nd), ramet biomass density, and ramet population density did not differ significantly between the base and middle of the dune (*P* > 0.05). However, they were significantly higher at the peak than at the middle and base (*P* < 0.001) (Figures 4(b), 4(c), and 4(d)). The ramet biomass density at the peak was approximately 1.6–2.5 times higher than that at the base on each sampling date (Figure 4(c)).

The aboveground biomass did not differ significantly between May 15th and June 2nd or between May 1st and June 23rd (Figure 4(a)). The underground biomass on June 2nd was significantly different from that on April 19th, May 1st, May 15th, and June 23rd, but there were no significant differences among these four sampling dates (Figure 4(b)). The ramet biomass density did not differ significantly among the dates April 19th, May 1st, May 15th, and June 23rd (*P* > 0.05) (Figure 4(c)). However, the ramet population density did not differ significantly among the sampling dates (*P* > 0.05) (Figure 4(d)).

### 3.4. Plasticity of Quantitative Characteristics of* C. physodes *at Different Locations on the Dune

**Figure 5:**
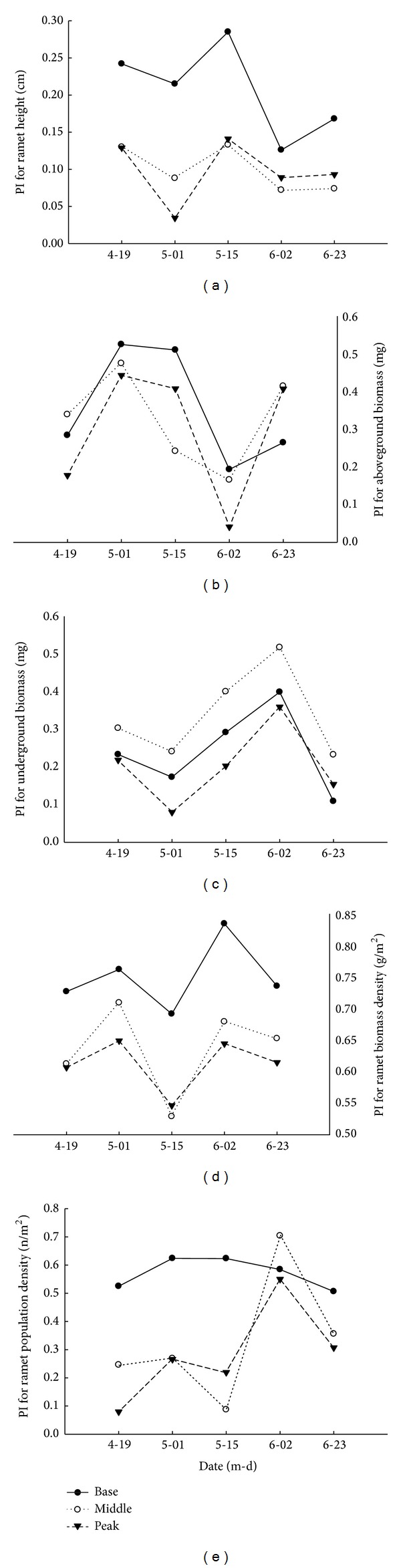
Plasticity index (PI) for ramet height, aboveground biomass, underground biomass, ramet biomass density, and ramet population density of* Carex physodes* on different sampling dates and locations of the sand dune.

The responsiveness of quantitative characteristics to differences in the location on the dune was compared for the sampling dates using the PI. The plasticity of the quantitative characteristics exhibited different patterns, and this phenotypic plasticity varied among the different sampling dates (Figure 5). The ramet height, aboveground biomass, ramet biomass density, and ramet population density showed higher plasticity at the base of the dune but lower plasticity at the peak of the dune. However, the underground biomass showed higher plasticity at the peak. The ramet height had the highest PI on May 15th. In contrast, the underground biomass, ramet biomass density, and ramet population density showed the highest PI on June 2nd.

### 3.5. The Linear Regression Relationship between Quantitative Characteristics of* C. physodes*

**Figure 6:**
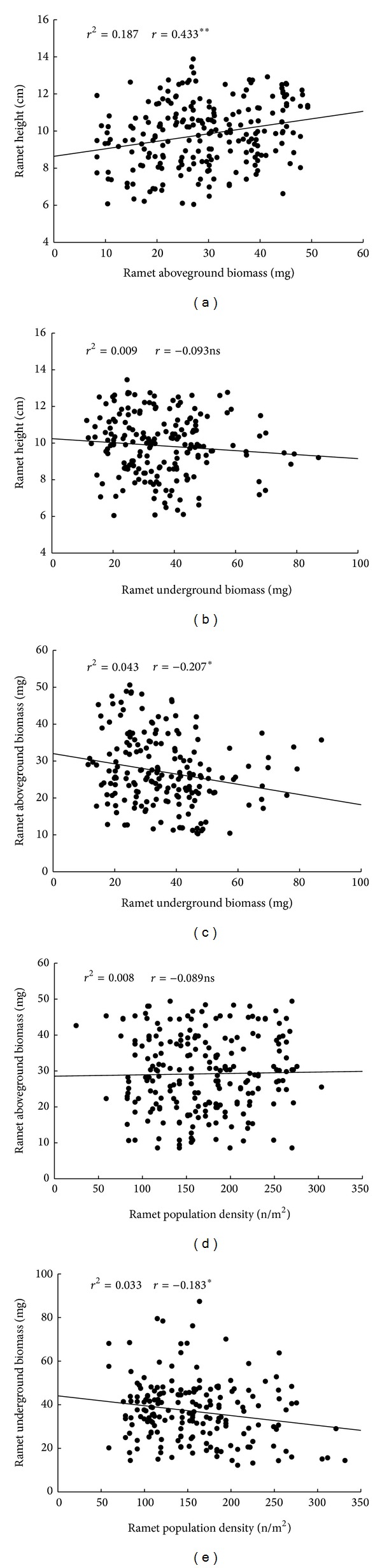
The linear regression between different quantitative characteristics of* Carex physodes* (**P < 0.01, *P < 0.05, and ns = no significant). Each point represents a separate ramet on different locations, *n* = 21.

The ramet height and aboveground biomass showed positive correlations (*P* < 0.01) (Figure 6(a)). The ramet aboveground biomass and ramet population density showed positive but not significant correlations (*P* > 0.05). However, the aboveground and underground biomasses, the underground biomass, and ramet population density showed significant negative correlations (*P* < 0.05) (Figures 6(c) and 6(e)). The ramet height and ramet underground biomass showed no significant negative correlation (Figure 6(d)).

## 4. Discussion

In the Gurbantunggut Desert, evaporation exceeds rainfall [6]. The average temperature and precipitation are 13.6°C and 24.7 mm, respectively, in May and 19.4°C and 19.1 mm, respectively, in April. However, the increasing temperatures, associated with the greater evaporation rates, cause an increased water loss and decreased input of water [18]. Therefore, soil water availability is the prime factor governing and limiting the size of the plants. In this study, the ramet height, aboveground biomass, and ramet population density gradually increased and reached their highest values in mid-May.* C. physodes* increased its branching density to make full use of the free space and sufficient water resources, and it gradually increased its biomass productivity in the spring, when water resources are sufficient. From mid-May to early June, moisture is depleted in the 5–20 cm sand layer, where the root system of* C. physodes* is primarily concentrated. The underground biomass and biomass density increased during this period. This observation is consistent with the ecological prediction that plants from competitive environments should increase their allocation to clonal propagation to escape poor environmental conditions [19].

Wang et al. and Zhang et al. studied the population characteristics of* Eremosparton songoricum* (Fabaceae) in different habitats. These researchers found that the underground biomass and biomass density in desert populations and natural bare sand areas were significantly higher than those in riverside and artificial sand-fixed areas and that the underground biomass and biomass density of* E. songoricum* increased as the soil water content decreased [20, 21]. The aboveground biomass, underground biomass, ramet biomass density, and ramet population density of* C. physodes* at the peak of the dune were significantly higher than those at the base and middle of the dune. These findings are consistent with the results of Wang et al. and Zhang et al. A possible explanation is that, at the base and middle, the soil resources are richer and the species richness is greater [22]. The peak of the dune is the location with the poorest resources (water and nutrition) [13]. Clonal integration may alleviate the competition-mediated stress on the ramets and enable the exploration of open and stressful habitats to better use resource patches and flourish in low-productivity habitats [23]. At the resource-poor dune peak,* C. physodes* increased its density to effectively transform water through clonal integration. The higher plant density and vigorous competition between neighboring plants at the base and middle of the dune may cause this species to improve its competitiveness by elongating the spacer length and by reducing the degree of branching frequency. Large amounts of well-developed biological soil crust, an important biological feature in the Gurbantunggut Desert that plays a key role in the functioning of the arid desert ecosystem, occur at the base and middle of the dune. The crust also prevents wind erosion and stabilizes and fertilizes the soil, thereby improving the growth of the ramet height of* C. physodes* [24].

Morphologically plastic species are capable of “foraging behavior”; they can modify the underground biomass and aboveground biomass to increase resource acquisition in response to changes in environmental conditions [25, 26]. The higher morphological plasticity found in the quantitative characteristics of the species at the base and middle of the dune was more closely associated with the soil moisture content. The favorable environment at the base and middle increased the plasticity of* C. physodes*. This phenomenon is also well-known for the temperature and water response of* Quercus ilex* [27]. Morphological plasticity in response to soil water plays an important role in resource acquisition in plants [28]. The linear regression analysis showed a positive aboveground biomass response to height but a negative underground biomass response to aboveground biomass and ramet population density. This species adjusts its biomass partitioning to equalize the growth limitations imposed by essential resources [29]. In dune habitat, the rhizomatous species of* Psammochloa villosa* (Gramineae) occupy considerable areas with large populations of ramets that are connected by rhizomes, it is beneficial to ramets to survive in the dune habitat with local environment stress as water deficiency [30]. Because root-related characteristics were more susceptible to water availability than leaf-related characteristics [31], this species allocated a higher proportion of its biomass underground (70%–98%) than aboveground (2%–30%). The research results of Maurer and Zedler are consistent with this phenomenon. In nutrients plentiful habitat,* Phalaris arundinacea* (Poaceae) maximized aboveground growth (nearly 75% more resources) to capture more light; and where nutrients are scarce, it increased belowground biomass (approximately 30% more resources) [32]. This distribution is vital for moderating the overall water (nutrients) loss through transpiration and increases the absorptive surface area exposed to the sandy habitat [33]. The aboveground and underground biomass, biomass density, and population density declined from early to late June, when the aboveground portions of the plant senesced and photosynthesis stopped.

In summary, the increasing temperatures exaggerated the existing water deficiency, which affects the growth and size of plants, especially ephemeral plants, a group of desert flora that are sensitive to a lack of soil water. Morphological plasticity is the most important feature of clonal plants that allows them to adjust adaptively to environmental heterogeneity on the dune. In contrast, clonal integration, represented by variation in biomass allocation, also greatly improves the ability of this rhizomatous species to tolerate water deficiency. Clonal integration may be an adaptive strategy of ephemeral rhizomatous plants growing in desert habitats with frequent disturbances, enabling these plants to become major contributors to dune surface stabilization and desert rehabilitation in the spring and early summer.

## Figures and Tables

**Table 1:** Three-way ANOVA of the effects of location, soil layer, sampling date, and their interactions on the soil moisture content.

Source	d.f.	SS	MS	*F*-value	*P* value
Location	2	4.244	2.122	22.595	0.000
Soil layer	3	54.176	18.059	192.284	0.000
Sampling date	4	45.504	11.376	121.129	0.000
Location × soil layer	6	2.052	0.342	3.642	0.002
Location × sampling date	8	2.301	0.288	3.064	0.004
Soil layer × sampling date	12	13.134	1.094	11.654	0.000
Location × soil layer × sampling date	24	1.926	0.080	0.855	0.661

**Table 2:** Two-way ANOVA of the effects of location, sampling date, and their interactions on the ramet height, aboveground biomass, underground biomass, ramet biomass density, and ramet population density.

	Source	d.f.	SS	MS	*F*-value	*P* value
Ramet height	Location	2	17.305	8.152	3.679	0.027
	Sampling date	4	276.826	66.957	30.219	0.000
	Location × sampling date	8	9.411	1.176	0.531	0.833

Aboveground biomass	Location	2	33.210	16.605	6.614	0.002
	Sampling date	4	69.240	17.310	6.895	0.000
	Location × sampling date	8	136.218	17.027	6.782	0.000

Underground biomass	Location	2	0.552	0.276	12.962	0.000
	Sampling date	4	1.847	0.462	21.693	0.000
	Location × sampling date	8	0.345	0.043	2.029	0.045

Ramet biomass density	Location	2	827867.708	41393.354	116.009	0.000
	Sampling date	4	18051.739	4512.935	12.648	0.000
	Location × sampling date	8	8128.117	1016.015	2.847	0.005

Ramet population density	Location	2	191020.398	95510.199	33.990	0.000
	Sampling date	4	11304.919	2826.230	1.006	0.412
	Location × sampling date	8	29459.721	4208.532	1.498	0.185
